# Editorial: Benzodiazepine addiction: from lab to street

**DOI:** 10.3389/fpsyt.2023.1287352

**Published:** 2023-09-22

**Authors:** Lais F. Berro, Thiago M. Fidalgo, James K. Rowlett, Vitor S. Tardelli

**Affiliations:** ^1^Department of Psychiatry and Human Behavior, Center for Innovation and Discovery in Addictions, University of Mississippi Medical Center, Jackson, MS, United States; ^2^Department of Psychiatry, Universidade Federal de São Paulo, São Paulo, Brazil; ^3^Translational Addictions Research Lab, Centre for Addiction and Mental Health, Department of Psychiatry, University of Toronto, Toronto, ON, Canada

**Keywords:** benzodiazepine, Z-drug, abuse, dependence, prescriptions

Benzodiazepine-type drugs (benzodiazepines and newer non-benzodiazepines, such as “Z-drugs”) are important therapeutic tools in psychiatry and general medicine. Despite their clinical usefulness, benzodiazepine-type drugs also are associated with several unwanted side effects, including abuse and dependence. In fact, the misuse and abuse of benzodiazepines have increased dramatically in recent years, with overdose deaths on the rise, especially with combinations of benzodiazepines and opioids (1). Given these concerns, the goal of this Research Topic was to highlight novel research examining factors related to the misuse, abuse, and dependence associated with benzodiazepine-type drugs. The success of this Research Topic, which includes 14 published manuscripts ranging from study protocols to review articles, emphasizes the growing interest and importance of this subject to the scientific community. This Research Topic spans multiple topics of investigation relating to benzodiazepine research, including pre-clinical studies, new epidemiology and novel treatment approaches.

Epidemiological studies published in this Research Topic emphasize that benzodiazepine use is on the rise worldwide. Coteur et al. reported an overall increase in benzodiazepine-type drug prescriptions between 2000 and 2019 in Flanders, Belgium. This was manifested as an increase in the number of male patients receiving three or more prescriptions at ages 18–44 and female patients over 65 years of age (Coteur et al.). McHugh et al. reported data on the prevalence of benzodiazepine and Z-drug misuse in the U.S. National Survey on Drug Use and Health from 2015 to 2019. According to their findings, 2% of the population was estimated to have misused a benzodiazepine in the past year, while <0.5% misused Z-drugs. Of note, studies in this Research Topic also corroborate the notion that benzodiazepine use increased due to the COVID-19 pandemic. Perelló et al. conducted a prospective observational study on benzodiazepine prescriptions in Catalonia from March 2020 to December 2021, showing an increase in benzodiazepine prescriptions during that period compared to the previous 2 years.

As evidenced by the studies by Coteur et al. and Perelló et al., the recent increase in benzodiazepine use is partially due to higher benzodiazepine prescription rates. Takeshima et al. reported that physicians are compelled to prescribe benzodiazepine-type drugs frequently despite rating these drugs as unsafe, often choosing efficacy over safety. In fact, the authors describe that physicians often opt to prescribe benzodiazepine drugs over other sleep aids rated as both safe and effective, such as orexin receptor antagonists (Takeshima et al.). These findings suggest that interventions are needed to reduce benzodiazepine prescription rates and, consequently, the public health burden of benzodiazepine use. To address this issue, Kinney et al. proposed the use of machine learning methods to develop algorithms to classify patients by their likelihood of receiving a benzodiazepine prescription and the number of benzodiazepine prescriptions they are likely to receive at a given patient-physician encounter. Their study showed that support-vector machine and random forest algorithms can accurately classify individuals who are at risk for receiving a benzodiazepine prescription (Kinney et al.), which could ultimately guide clinical practice.

Several other factors also can influence benzodiazepine use. Zandonai et al. describe clinical cases of elite endurance athletes reporting benzodiazepine use to manage insomnia, pain, and to speed up recovery. Of note, the authors emphasize that sports medicine physicians are often unaware of the dangers associated with chronic benzodiazepine use, and benzodiazepine prescription and tapering guidelines are discussed (Zandonai et al.). As part of their physician guidelines, the authors emphasize the need to taper the benzodiazepine dosage while introducing an alternative therapy. In accordance with the International Patient Decision Aid Standards, Aoki et al. developed a decision aid for individuals with anxiety disorders to help with decision-making regarding discontinuation of benzodiazepine treatment. The goal of their approach was to aid patients and healthcare providers in determining whether or not to taper off of benzodiazepines and, if tapering, whether or not to implement cognitive behavioral therapy for anxiety during tapering (Aoki et al.). Their decision aid was well-accepted by both patients and physicians, and could become an important clinical tool.

Greenwald et al. also reported that anhedonia (positive-affective deficit) predicted increased benzodiazepine demand in past-year benzodiazepine users receiving treatment for opioid use disorder. Anhedonia also predicted opioid demand, emphasizing that deficits in the experience and anticipation of reward seem to influence the use of these drugs (Greenwald et al.). In addition to clinical studies, a pre-clinical investigation by Jovita-Farias et al. investigated the relationship between different behavioral effects of the benzodiazepine midazolam in male mice, demonstrating that midazolam preference (i.e. reward) is a multifactorial behavior, and is not dependent solely on the emergence of therapeutic (anxiolytic-like) effects, learning impairments, or on genetic factors. Together, these findings suggest that many factors can interact to influence the decision to use benzodiazepines, both clinically and recreationally, and that further studies are needed to determine factors contributing to benzodiazepine use. To address this gap, Zamboni et al. propose a study protocol using virtual reality to assess the impact of benzodiazepine-associated environmental cues on patient-reported benzodiazepine craving and affective states.

A common theme across several of the publications in this Research Topic was the investigation of Z-drug use as a potential emerging problem. Coteur et al. reported that while alprazolam was the most largely prescribed benzodiazepine in 2000, by 2019 zolpidem had become the most largely prescribed benzodiazepine-type drug in Flanders, Belgium. On the other hand, McHugh et al. reported that Z-drug misuse in the U.S. was less common than benzodiazepine misuse, and those reporting Z-drug misuse presented less concurrent substance use and lower clinical severity. In agreement, Campagnari et al. showed that the use of high doses of zolpidem was not associated with adverse cardiovascular effects (specifically, corrected QT interval elongation), suggesting that zolpidem is a safe drug even when used at higher than recommended doses. Furthermore, Koniuszewski et al. screened the publicly available U.S. FDA adverse event reporting system database for benzodiazepine-type drugs, and their findings suggest that benzodiazepines and Z-drugs differ vastly in adverse event profiles, with benzodiazepines showing a higher incidence of adverse events. Together, these findings suggest that while Z-drug prescription is on the rise, Z-drugs may be safer than conventional benzodiazepines. Further research is necessary to conclusively determine the clinical implications of long-term Z-drug use and misuse.

Of note, Koniuszewski et al. also reported significant sex differences in the rates of adverse events reported for benzodiazepine-type drugs. Specifically, neuropsychiatric adverse events observed for conventional benzodiazepines were more prevalent in females than in males (Koniuszewski et al.). While not directly investigated in their study, the authors emphasize the possibility that steroid hormones may influence the emergence of benzodiazepine-induced adverse events. In fact, Cook et al. demonstrated that, in contrast to their previous study with males, combinations of the conventional benzodiazepine triazolam and the neuroactive steroid pregnanolone induced synergistic reinforcing and sedative effects in female rhesus monkeys. These results corroborate the notion that sex differences exist in benzodiazepine-neuroactive steroid combinations, which could contribute to the different side effect profiles reported between sexes in the study by Koniuszewski et al..

Finally, the thorough review by Engin discussed the mechanisms underlying the abuse/misuse-related effects of benzodiazepine-type drugs. The author reviewed studies suggesting that α1-containing GABA_A_ receptors may play an important role in benzodiazepine reinforcement, tolerance and dependence. The findings summarized in this review highlight the progress in the field of benzodiazepine research, yet also emphasize the need for further, systematic investigations elucidating the mechanisms underlying benzodiazepine misuse, abuse and dependence. For instance, zolpidem, the most widely prescribed Z-drug (Coteur et al.; McHugh et al.), has selective affinity for α1-containing GABA_A_ receptors, which, according to the review by Engin, would predict a higher potential for abuse compared to conventional benzodiazepines. However, the study by McHugh et al. shows that, in the U.S., Z-drug misuse is less prevalent than benzodiazepine misuse. Together, these findings suggest that other abuse-related mechanisms also may be at play, including other GABA_A_ receptor subtypes (e.g., α2-containing GABA_A_ receptors, see Engin), and highlight the need for further cross-talk between pre-clinical and clinical researchers.

## Author contributions

LB: Writing—original draft, Writing—review and editing. TF: Writing—review and editing. JR: Writing—original draft, Writing—review and editing. VT: Writing—review and editing.
